# An N-Shaped Association between Population Density and Abdominal Obesity

**DOI:** 10.3390/ijerph19159577

**Published:** 2022-08-04

**Authors:** Bindong Sun, Xiajie Yao, Chun Yin

**Affiliations:** 1 The Center for Modern Chinese City Studies, East China Normal University, Shanghai 200241, China; 2 Research Center for China Administrative Division, East China Normal University, Shanghai 200241, China; 3 Institute of Eco-Chongming, 20 Cuiniao Rd., Chenjia Zhen, Chongming, Shanghai 202162, China; 4 School of Urban and Regional Science, East China Normal University, Shanghai 200241, China; 5 Future City Laboratory, East China Normal University, Shanghai 200241, China

**Keywords:** neighborhood design, residential density, obesity, healthy city, nonlinear effect

## Abstract

Abdominal obesity is a threat to public health and healthy cities. Densification may reduce abdominal obesity, but current evidence of the relationship between population density and abdominal obesity is not conclusive. The aim of this study was to disentangle the nonlinear association between population density and abdominal obesity. Data came from the 2004–2015 China Health and Nutrition Survey, which included 36,422 adults aged between 18 and 65 years. Generalized additive models (GAMs) were applied to explore how population density was associated with objectively measured waist circumference (WC) and waist-to-height ratio (WHtR), after controlling for other built environmental attributes, socioeconomic characteristics, and regional and year fixed effects. We found that population density had N-shaped associations with both WC and WHtR, and the two turning points were 12,000 and 50,000 people/km^2^. In particular, population density was positively correlated with abdominal obesity when it was below 12,000 people/km^2^. Population density was negatively associated with abdominal obesity when it was between 12,000 and 50,000 people/km^2^. Population density was also positively related to abdominal obesity when it was greater than 50,000 people/km^2^. Therefore, densification is not always useful to reduce abdominal obesity. Policy-makers need to pay more attention to local density contexts before adopting densification strategies.

## 1. Introduction

Obesity is a serious threat to global public health because it is a major risk factor for both coronavirus disease and many noncommunicable diseases [1,2]. Based on the distribution of fat in the body, obesity can be classified into general obesity and abdominal obesity. Although general obesity measured by body mass index is related to reduced life expectancy [3], abdominal obesity measured by waist circumference and waist circumference-related measures is a better indicator than general obesity to predict type 2 diabetes and cardiovascular diseases [4]. Moreover, due to differences in lifestyles and genes, the prevalence rates of abdominal obesity (37.6%) are much higher than those of general obesity (15.0%) in China [5]. Hence, preventing and reducing abdominal obesity is important for obesity control.

In American and European countries, scholars recommend densification of the residential environment to prevent and reduce general obesity [6,7]. However, they have given less attention to abdominal obesity, which is a severe obesity problem in Asian countries [8]. Only a few studies have explored the relationship between densification (measured by population density) and abdominal obesity, and their findings are inconclusive. First, several studies assumed that population density had a linear association with abdominal obesity, but they observed opposite linear relationships. In particular, a study found a positive association of population density with abdominal obesity in rural China [9], whereas another found an adverse relationship of population density with abdominal obesity in urban areas in France [10]. Moreover, several studies assumed that population density had nonlinear associations with abdominal obesity. However, their findings were also the opposite. In particular, they observed inverted U-shaped and U-shaped curves between population density and abdominal obesity in urban UK and urban Chinese areas, respectively [11,12]. Overall, the relationship between population density and abdominal obesity is still not well understood.

To bridge the research gap, the goal of this study is to answer two research questions. Does population density have a nonlinear relationship with abdominal obesity? If so, does the nonlinear relationship follow a specific pattern, or is it irregular? To answer these questions, we applied generalized additive models (GAMs) to the China Health and Nutrition Survey dataset between 2004 and 2015, which included 36,422 adults across 15 provinces in China, to explore whether population density has a nonlinear relationship with abdominal obesity.

## 2. Materials and Methods

### 2.1. Data

Data were obtained from the China Health and Nutrition Survey (CHNS). This survey has been running since 1989 and the last data were collected in 2018. During these 29 years, eleven waves of data have been collected by the University of North Carolina at Chapel Hill in collaboration with the China Center for Disease Control and Prevention. This survey employed a multistage, random cluster sampling method to select respondents. First, 15 provincial-level divisions were selected, covering different economic and cultural regions in mainland China. Second, four counties and two cities were selected within each provincial-level division based on levels of economic development. Third, 330 communities (210 in villages/townships and 120 in urban/suburban neighborhoods) were selected within the selected counties and cities. Fourth, face-to-face interviews were conducted with residents to collect individual data, and community officials were interviewed to collect community data. More information related to the CHNS dataset is available at https://www.cpc.unc.edu/projects/china (accessed on 19 June 2022).

This study used the CHNS data of adult respondents aged between 18 and 65 years, collected in 2004, 2006, 2009, 2011, and 2015. This is because most built environmental variables were collected starting in 2004, and the data from 2018 were unavailable. After excluding the observations with missing values, the working sample in this study comprised 36,422 observations.

### 2.2. Abdominal Obesity

Abdominal obesity was the dependent variable in this study. It was reflected by waist circumference (WC) and waist-to-height ratio (WHtR), which were objectively measured by professional staff according to the standardized measurement process required by the China Obesity Working Group [13]. WC is a proxy for the degree of abdominal fat accumulation [14]. It was measured at the midpoint of the mid-axillary line between the iliac crest and the lower border of the rib cage in a horizontal circumference in centimeters by using an inelastic tape [15]. WHtR is defined as the ratio of WC to height, which is another valid proxy of abdominal obesity and is a better indicator of the risk of obesity-related diseases than the waist-to-hip ratio [16].

### 2.3. Community Built Environmental Attributes

The scopes of communities are based on the administrative boundaries of neighborhoods/villages. Population density was the key independent variable, which is a proxy of compactness. Population density refers to the population in 1000 people divided by the community area in square kilometers. Note that compared to other density measures related to population (e.g., dwelling density and household density), population density is more accurate because it considers household size and vacancy status [17].

Following previous studies [18,19,20], some other built environmental attributes were treated as covariates in this study because they were correlated with both population density and obesity. Business density was measured by the number of private businesses divided by the community area. Fast food restaurant density was measured by the number of Western fast-food restaurants divided by the area of the community. Distances from the community to the nearest wet market, park, school, and bus stop were self-reported by community officials.

### 2.4. Individual Socioeconomic Characteristics

Individual socioeconomic attributes were also covariates in this study, including sex (men vs. women), age (in years), nationality (Han Chinese vs. others), urban status (urban residents vs. rural residents), marital status (married vs. others), education (in years), employment status (farmers (reference group); nonfarmers; unemployed), household income (in 10,000 yuan/year), and household size (i.e., the number of household members living in the same household). Moreover, covariates included regional effects (i.e., the surveyed province) and time effects (i.e., the surveyed year).

### 2.5. Analytical Approaches

This study employed generalized additive models (GAMs) to investigate the potential nonlinear relationship between population density and abdominal obesity. The GAM comprises two parts, which are parametric and nonparametric components [21]. The parametric component uses the traditional linear regression approach, which is used to estimate the linear association between independent variables and the dependent variable [22]. The nonparametric component relaxes the linear assumption and uses smooth functions to estimate the nonlinear association of the independent variable with the dependent variable [23]. Overall, the GAM is a semiparametric model that can estimate not only linear associations but also nonlinear associations between independent variables and the dependent variable.

GAMs have several advantages. First, compared to traditional linear models, they allow scholars to explore complex and irregular nonlinear relationships between independent variables and the dependent variable through smooth functions [24]. Second, compared to machine learning approaches, they are more interpretable and provide the significance of coefficients [23].

To explore the nonlinear relationship between population density and abdominal obesity, we set the GAMs as follows:

(1) $$AO_{i}=s\left(PD_{i}\right)+\beta_{BE}BE_{i}+\beta_{SES}SES_{i}+\beta_{RE}Regional_{i}+\beta_{YE}Year_{i}+\varepsilon_{i}$$

where *AO_i_* refers to the abdominal obesity of an individual *i*, which is measured by WC or WHtR. Given that both WC and WHtR were continuous variables and followed normal distributions, we used Gaussian with identity link function in the models. s(*PD)* was the nonparametric component of the model. *PD* was population density and *s(PD)* indicated population density fitted by a smooth function *s(.)*. In this study, we used thin-plate splines to fit the potential nonlinear relationship of population density with abdominal obesity. Other variables in the model were covariates and were treated as the parametric component, including *BE*, *SES*, *Regional*, and *Year*, which represented the matrix of other built environmental attributes, socioeconomic characteristics, regional, and year fixed effects, respectively. The GAM used a linear process to estimate the average effects of these variables on abdominal obesity. Notably, we tested intraclass correlations of WC and WHtR in multilevel null models. The results showed that intraclass correlations were below 0.1 in both models, suggesting that the spatial dependency at the community level was trivial [25,26]. Hence, it was not necessary to consider the random effect at the community level. All analyses were performed using the “mgcv” package in R [27].

## 3. Results

### 3.1. Descriptive Analysis

Table 1 presents the characteristics of the respondents and communities. The mean WC and WHtR were 82.46 cm and 0.51, respectively. Men accounted for 47%. The mean age was 45 years old. Han Chinese, urban, and married residents accounted for 88%, 35%, and 87%, respectively. The mean number of years of education was approximately 9 years, which is equivalent to a high school education. Unemployed residents, farmers, and nonfarmers accounted for 35%, 24%, and 41%, respectively. On average, annual household income was approximately 48 thousand yuan. Each household, on average, had four household members living together.

In terms of built environmental attributes, the mean population density was 6420 people/km^2^. There were four business companies in a community on average, and the mean density of fast-food restaurants was 0.7 restaurants/km^2^. The distances from the community to the nearest wet market, park, school, and bus stop were 1.4 km, 8.6 km, 0.6 km, and 1.1 km, respectively.

### 3.2. Associations of Population Density and Covariables with WC and WHtR

Table 2 presents the results of the GAMs after adjusting for regional and year fixed effects. The adjusted R^2^ values were 0.16 and 0.14 in the WC and WHtR models, respectively. The adjusted R^2^ values in the present study were higher than those in previous studies, which were often below 0.1. A possible reason is that our study relaxed the assumption of linearity by considering the nonlinear association of population density with WC and WHtR, and hence, the goodness-of-fit of the models was better.

GAMs use effective degrees of freedom (edf) to present the degree of nonlinearity in the association of population density with abdominal obesity. In the both WC and WHtR models, the edf values of population density were 3.870 (*p* < 0.001) and 3.423 (*p* < 0.001), respectively. These findings imply that population density had significant nonlinear relationships with both WC and WHtR.

**Figure 1:**
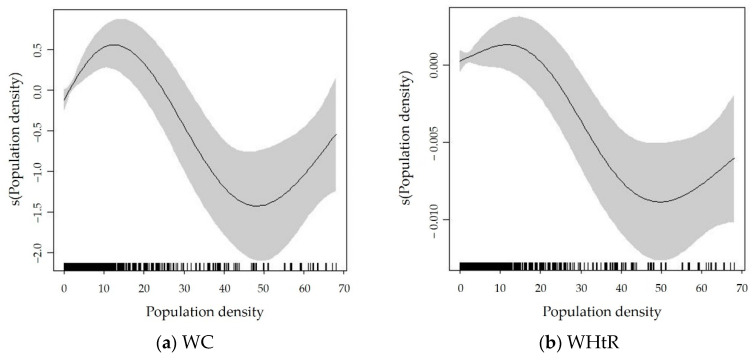
Nonlinear relationships between population density and abdominal obesity based on pooled data from 2004 to 2015 (**a**) WC; (**b**) WHtR. The *x*-axis represents the exact value of population density and the *y*-axis represents the estimation results of population density based on the smooth function. The shaded areas indicate 95% confidence intervals. The tick marks above the *x*-axis represent the number of observations at that value.

Figure 1 illustrates that population density had nonlinear associations with both WC and WHtR, and the pattern was an N-shaped relationship. First, when the population density was between 0 and 12,000 people/km^2^, it was positively related to WC and WHtR. However, the slope of population density on WHtR was much flatter than that on WC. Second, when the population density was between 12,000 and 50,000 people/km^2^, it had a negative association with WC and WHtR. In this range, the slopes of population density on WC and WHtR were similar. Third, when the population density was above 50,000 people/km^2^, it again had a positive relationship with WC and WHtR. A slight difference was that the slope of population density on WHtR was slightly flatter than that on WC in the same range. That is, a higher population density tended to first lead to an increase in WC and WHtR, then to a decrease in WC and WHtR, and again to an increase in WC and WHtR after the threshold.

Table 2 also presents the results of the parametric analysis of the WC and WHtR models. Both business density and fast-food restaurant density had positive relationships with WC and WHtR. However, distance to the nearest park had negative associations with WC and WHtR. We did not find any significant association of other built environmental attributes with WC and WHtR. Most socioeconomic covariates were significantly related to both WC and WHtR. In particular, men were more likely to have a larger WC and a lower WHtR. Age had positive relationships with both WC and WHtR. Han Chinese residents were more likely to have a larger WC. Married and poorly educated residents tended to have a larger WC and higher WHtR. Compared to farmers, nonfarmers and unemployed residents had a greater likelihood of having a larger WC and higher WHtR. Household size was only negatively associated with WC. We did not find significant associations of urban/rural areas and household income with either abdominal obesity measure.

### 3.3. Robustness Check

To check whether the N-shaped association of population density with abdominal obesity was robust, we used cross-sectional data collected in 2015 to examine the results of baseline models. Table 3 presents the model results after controlling for other built environmental elements, socioeconomic characteristics, and regional fixed effects. In both WC and WHtR models, the edf values of population density were 3.969 (*p* < 0.001) and 3.519 (*p* < 0.01), respectively, suggesting that population density was nonlinearly related to abdominal obesity.

**Figure 2:**
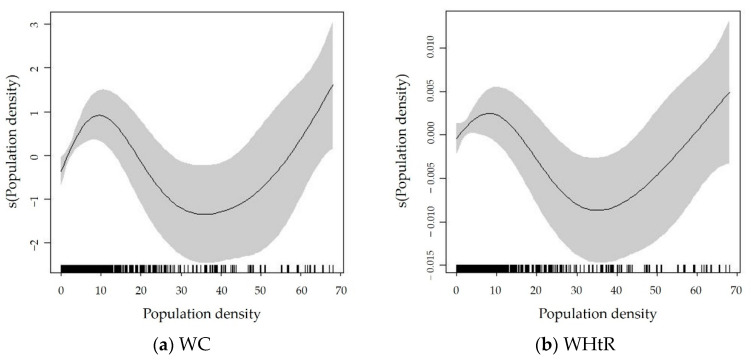
Nonlinear relationships between population density and abdominal obesity based on 2015 cross-sectional data (**a**) WC; (**b**) WHtR. The *x*-axis represents the exact value of population density and the *y*-axis represents the estimation results of population density based on the smooth function. The shaded areas indicate 95% confidence intervals. The tick marks above the *x*-axis represent the number of observations at that value.

Figure 2 shows that population density has N-shaped associations with both WC and WHtR, which is in line with the findings based on the pooled models. This suggests that the N-shaped association between population density and abdominal obesity was robust. In particular, when population density was between 0 and 12,000 people/km^2^, population density was positively related to both WC and WHtR in 2015. When population density was between 12,000 and 37,000 people/km^2^, it had negative associations with both WC and WHtR in 2015. This turning point was slightly lower than that (50,000 people/km^2^) in the pooled models. Beyond 37,000 people/km^2^, population density had positive associations with both WC and WHtR. Overall, the general pattern of the effect of population density on abdominal obesity was an N-shaped curve in both pooled models (data between 2004 and 2015) and cross-sectional models (data in 2015).

## 4. Discussion

Based on pooled cross-sectional data across 15 provinces in China between 2004 and 2015, this study applied GAMs to explore the irregular nonlinear association between population density and abdominal obesity after controlling for other built environmental attributes, respondents’ socioeconomic characteristics, and regional and year fixed effects. The results show that population density had an N-shaped relationship with abdominal obesity measured by WC and WHtR. Moreover, the N-shaped relationship had two important thresholds: 12,000 and 50,000 people/km^2^.

**Figure 3:**
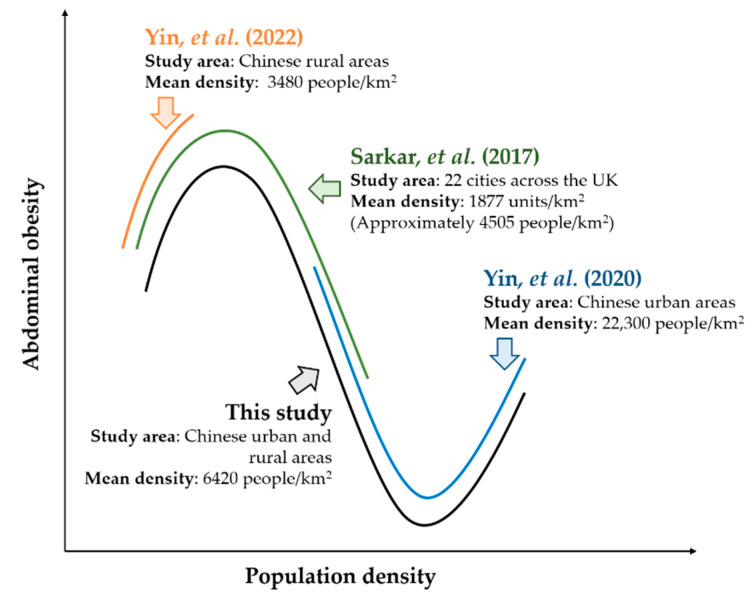
Associations of population density with abdominal obesity in the literature and the current study [9,11,12].

Our finding of an N-shaped relationship contributes to the literature by integrating the mixed findings in previous studies (Figure 3). In the low-density context, Yin, Yao, and Sun [9] found that population density was positively associated with adults’ WHtR in rural China, which had a mean population density of 3480 people/km^2^. Their result is supported by the left side of the N-shaped relationship. In the low- and medium-density context, Sarkar, Webster, and Gallacher [11] found an inverted U-shaped association between dwelling density and adults’ WCs based on the UK Biobank dataset with a mean residential density of 1877 units/km^2^ (approximately 4505 people/km^2^). Their finding is in line with the left and middle parts of the N-shaped association. Moreover, in the medium- and high-density context, they observed that population density had a U-shaped relationship with adults’ waist-to-hip ratios in Chinese urban areas with a mean population density of 22,300 people/km^2^. This finding is consistent with the middle and right parts of the N-shaped association.

**Figure 4:**
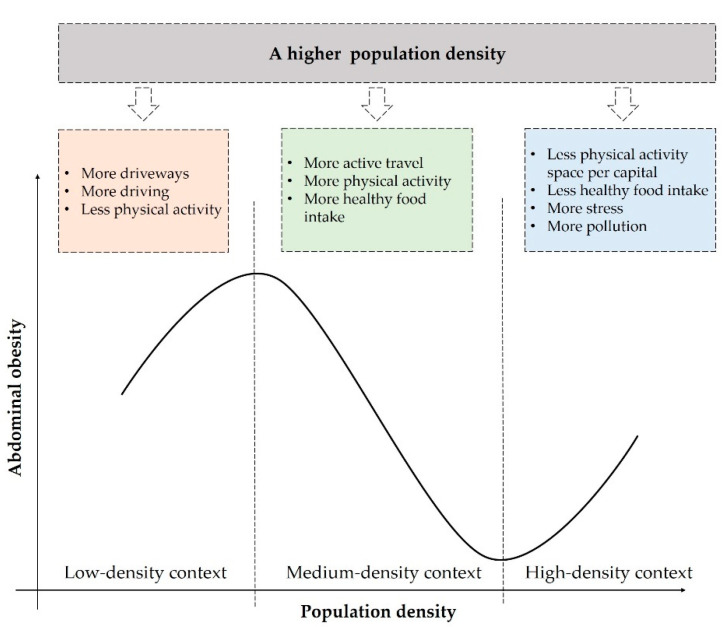
The conceptual framework of the N-shaped relationship between population density and abdominal obesity.

The N-shaped curve offers a more complete framework to interpret the relationship between population density and abdominal obesity (Figure 4). Although there are many pathways from population density to abdominal obesity, the N-shaped association is their trade-off result. That is, at a certain range of population density, some pathways are overwhelmed by the others.

In the low-density context, with a population density below 12,000 people/km^2^, the risk of abdominal obesity rises as population density increases. This is consistent with findings in rural areas [28]. First, a higher population density in the low-density context has a positive influence on abdominal obesity by increasing car ownership and driving [29,30]. Although a higher population density meets the minimum population thresholds of having daily destinations and facilities (e.g., supermarkets), destination accessibility is still low without cars in low-density areas [31]. Given that more destinations stimulate people’s travel demand, people living in low-density contexts tend to own more cars and drive to daily destinations [32]. Many studies have confirmed that driving is positively related to sedentary behavior and physical inactivity, leading to a higher risk of abdominal obesity [30,33]. Moreover, a higher population density in a low-density context tends to reduce rural residents’ physical activity by decreasing outdoor open spaces, leading to a higher risk of being obese [28].

In the medium-density context, with a population density between 12,000 and 50,000 people/km^2^, a higher population density has a negative relationship with abdominal obesity. This is consistent with most previous studies conducted in Western cities [34,35], which shows that the risk of being obese tends to be lower with the increase in population density. Moreover, a higher population density is positively related to active travel and is negatively associated with driving [36], leading to more transport-related physical activities and a lower likelihood of being obese [37]. On the other hand, in this density range, a higher population density brings more healthy food stores and improves the availability of fresh vegetables and fruits [38], which is important to promote healthy diets and to reduce obesity [39,40].

In the high-density context, with a population density above 50,000 people/km^2^, the risk of abdominal obesity increases with increased population density. This is consistent with previous findings from Chinese megacities [41,42]. First, a higher population density in the high-density context is related to fewer green spaces and sport facilities per capita, resulting in higher levels of physical inactivity and increased obesity risks [43]. Second, residents often live in a food swamp if the community has a high population density. In a food swamp, most of the food for sale is less nutritious and high in calories, such as bubble tea and fried chicken [44]. Such contexts are common in high-density Chinese communities and tend to increase obesity by increasing the likelihood of high-calorie dietary intake [45]. Third, people living in densely populated areas are more likely to suffer from stress, insomnia, and social withdrawal, which are important contributors to obesity [46]. Fourth, population density is positively related to air and noise pollution [36], which directly contribute to obesity [47].

It is worth mentioning that there are several possible reasons supporting the finding of an N-shaped relationship between population density and abdominal obesity in this study. First, previous studies usually assumed that population density had a linear relationship or a specific form (e.g., a quadratic function) with abdominal obesity. Therefore, they may have observed only restrictive associations. However, the present study employed GAMs, which do not require the relationship to follow any pre-assumption [23]. Hence, we can find a more realistic and nonlinear association of population density with abdominal obesity. Second, previous studies often focused on a specific context (e.g., a single city or urban/rural areas), which may have limited variations in population density. However, our national dataset covers communities in both low-density rural and high-density urban areas in China, with densities ranging from 1 person/km^2^ to 68,000 people/km^2^, which can help us observe the relationship of population density with abdominal obesity more comprehensively.

Additionally, we found that other built environmental attributes and individual socioeconomic characteristics were significantly related to abdominal obesity. Fast food restaurant density was positively related to abdominal obesity, which is in line with the literature [48]. This is possibly because a higher fast food restaurant density encourages residents to consume unhealthy foods and increases the risk of abdominal obesity. In contrast to previous studies, we found that both business density and park accessibility were positively related to abdominal obesity. These findings may be related to the Chinese context. Although we controlled for household income to capture the effects of individual economic status, built environmental attributes, such as business density and park accessibility, may still be related to the economic levels of individuals and communities (e.g., property). In China, rich people tend to live in communities with high business density and park accessibility for jobs, housing, and work–life balance. However, these rich people often misunderstand the potbelly to be a symbol of health and power due to a lack of health literacy, leading to the pursuit of abdominal obesity [49]. In terms of individual socioeconomic attributes, respondents who were older, Han Chinese, married, poorly educated, not farmers, and unemployed were more likely to be abdominally obese, which is consistent with previous studies [8,50]. An interesting finding was that sex had different associations with WC and WHtR. WC was higher among men than among women. This makes sense because of the differences in body structure between men and women. According to the WHO guidelines, a WC greater than or equal to 94 cm for men and greater than or equal to 80 cm for women is considered abdominal obesity [14]. After considering individual heights, we found that men had a lower WHtR than women, which is in line with previous studies [50].

To the best of our knowledge, this is the first study to explore and identify an N-shaped relationship between population density and abdominal obesity. The N-shaped relationship reminds planners to carefully consider the local density contexts when adopting densification and decentralization strategies to prevent and reduce obesity. Blindly following densification strategies recommended by American and European countries without focusing on the local context may not be conducive to residents’ health. In particular, in the low-density context, planners need to note that a slight increase in population density may not reduce obesity. Instead, it may increase the risk of obesity by promoting driving and physical inactivity. Hence, to prevent residents from becoming obese, it is essential to improve population density in the low-density context to a certain threshold, which can support residents’ travel to daily destinations by walking or cycling. In the medium-density context, an increase in population density is useful to reduce obesity, as it improves the accessibility of destinations and promotes both transportation-related and recreational physical activity. In the high-density context, decentralization strategies are necessary to prevent the negative externalities of megacities (e.g., pollution and crowding), which is also important to reduce abdominal obesity.

This study has several limitations due to the unavailability of data. First, we cannot infer any causality based on the pooled cross-sectional design. Future studies may test our N-shaped associations based on more rigorous research designs (e.g., randomized controlled trials). Second, the thresholds of the N-shaped association in this study may not be generalizable to other contexts. We encourage scholars to examine the thresholds of the N-shaped association in other countries and subpopulations. Additionally, if the data are available, we highly recommend that scholars conduct a global comparison study to confirm the N-shaped association. Third, the research data were collected between 18 and 7 years ago, which may not reflect the association between population density and abdominal obesity during the pandemic. Future studies may compare whether the N-shaped association still holds in the post-pandemic era. Fourth, the models omitted several covariates (e.g., health promotion strategies and self-care technologies). If these data are available in the future, scholars may adjust for them to improve the estimation accuracy of the association between population density and abdominal obesity.

Nevertheless, this study has several distinct strengths. First, compared with previous studies that assumed a linear or specific relationship between population density and abdominal obesity, we used GAMs to relax the traditional linear assumption and did not suppose any assumptions of the relationship. Hence, the results are more realistic and objective. Second, compared to previous studies focused on a specific context, the CHNS data covered 15 Chinese provinces, including low-, medium-, and high-density communities in urban and rural areas, which provides a more comprehensive density spectrum, allowing us to observe a more complete association between population density and abdominal obesity. Third, compared to self-reported obesity measures, the CHNS employed objective measures, which are more accurate and unbiased.

## 5. Conclusions

Population density plays an important role in shaping obesity. However, the literature does not provide clear evidence on the relationship between population density and abdominal obesity, particularly with regard to the complex and nonlinear influence of population density. Using GAMs with a national sample of 36,422 adults collected by the CHNS between 2004 and 2015, we found a clear N-shaped relationship between population density and abdominal obesity. That is, as population density increases, the risk of abdominal obesity undergoes a fluctuating process of increasing, then decreasing, and then increasing again. This is the first study that found an N-shaped relationship between population density and abdominal obesity, and it contributes to the literature by integrating the mixed results from previous studies into a more comprehensive theoretical framework. Therefore, although optimizing population density is a key strategy for planners and policy-makers to prevent and reduce abdominal obesity, they need to give more attention to local density contexts and identify whether densification is an appropriate policy.

## Figures and Tables

**Table 1:** Descriptive statistics of variables (sample size = 36,422).

Variable	Mean/%	Std. Dev.	Min	Max
Dependent Variables				
WC (cm)	82.46	10.56	45	107.30
WHtR	0.51	0.06	0.36	0.68
Built environmental attributes				
Population density (1000 people/km^2^)	6.42	13.59	0.001	68
Business density (count/km^2^)	3.93	17.95	0	150
Fast food restaurant density (count/km^2^)	0.68	3.41	0	26
Distance to the nearest wet market (km)	1.44	3.16	0	35
Distance to the nearest park (km)	8.57	15.08	0	90
Distance to the nearest school (km)	0.56	1.16	0	6
Distance to the nearest bus stop (km)	1.06	2.91	0	18
Socioeconomic characteristics				
Men	47%	—	—	—
Age (years)	45.49	12.19	18	65
Han Chinese	88%	—	—	—
Urban	35%	—	—	—
Married	87%	—	—	—
Years of education	8.60	3.96	0	18
Employment status				
Farmer	24%	—	—	—
Nonfarmer	41%	—	—	—
Unemployed	35%	—	—	—
Household income (10,000 yuan/year)	4.78	7.86	0	480
Household size (count)	3.75	1.53	1	13

**Table 2:** Results of the GAMs based on pooled data from 2004 to 2015 (sample size = 36,422).

Variable	WC		WHtR	
**Nonparametric Component**	**Edf**	**F**	**Edf**	**F**
Population density	3.870 ***	8.755	3.423 ***	8.037
**Parametric Component**	**Beta**	**SE**	**Beta**	**SE**
Other built environmental attributes				
Business density	0.01086 ***	0.00298	0.00005 *	0.00002
Fast food restaurant density	0.05282 **	0.01643	0.00025 *	0.00010
Distance to the nearest wet market	−0.01907	0.01944	−0.00011	0.00012
Distance to the nearest park	−0.02553 ***	0.00392	−0.00014 ***	0.00002
Distance to the nearest school	−0.04925	0.04956	−0.00040	0.00030
Distance to the nearest bus stop	0.02204	0.01907	0.00018	0.00012
Socioeconomic characteristics				
Men	4.35229 ***	0.10600	−0.00659 ***	0.00064
Age	0.16084 ***	0.00522	0.00135 ***	0.00003
Han Chinese	0.53568 **	0.18600	0.00055	0.00112
Urban	−0.08320	0.12289	−0.00112	0.00074
Married	1.67396 ***	0.16417	0.00868 ***	0.00099
Years of education	−0.13983 ***	0.01645	−0.00159 ***	0.00010
Employment status (ref. = farmers)				
Nonfarmers	1.59588 ***	0.15582	0.00729 ***	0.00094
Unemployed	1.70086 ***	0.15185	0.00889 ***	0.00092
Household income	−0.00082	0.00708	−0.00003	0.00004
Household size	−0.12949 ***	0.03747	−0.00043	0.00023
Region effects	Controlled	Controlled	Controlled	Controlled
Time effects	Controlled	Controlled	Controlled	Controlled
Goodness-of-fit				
Adjusted R^2^	0.162		0.144	

Note: * *p* < 0.05, ** *p* < 0.01, *** *p* < 0.001.

**Table 3:** Results of the GAMs based on 2015 cross-sectional data (sample size = 8690).

Variable	WC		WHtR	
**Nonparametric Component**	**Edf**	**F**	**Edf**	**F**
Population density	3.969 ***	5.038	3.519 **	3.347
**Parametric Component**	**Beta**	**SE**	**Beta**	**SE**
Other built environmental attributes	Controlled	Controlled	Controlled	Controlled
Socioeconomic characteristics	Controlled	Controlled	Controlled	Controlled
Region effects	Controlled	Controlled	Controlled	Controlled
Goodness-of-fit				
Adjusted R^2^	0.131		0.123	

Note: ** *p* < 0.01, *** *p* < 0.001.

## Data Availability

The individual data presented in this study are openly available at https://www.cpc.unc.edu/projects/china (accessed on 28 June 2022). Restrictions apply to the availability of community data, which are available with the permission of the Carolina Population Center.
